# Constructing an integrated genetic and epigenetic cellular network for whole cellular mechanism using high-throughput next-generation sequencing data

**DOI:** 10.1186/s12918-016-0256-5

**Published:** 2016-02-20

**Authors:** Bor-Sen Chen, Cheng-Wei Li

**Affiliations:** Department of Electrical Engineering, Lab. of Control and Systems Biology, National Tsing Hua University, Hsinchu, Taiwan

**Keywords:** HIV, miRNA, Epigenetic regulation, Integrated genetic and epigenetic cellular network, Next-generation sequencing data, Epigenetic cellular mechanisms

## Abstract

**Background:**

Epigenetics has been investigated in cancer initiation, and development, especially, since the appearance of epigenomics. Epigenetics may be defined as the mechanisms that lead to heritable changes in gene function and without affecting the sequence of genome. These mechanisms explain how individuals with the same genotype produce phenotypic differences in response to environmental stimuli. Recently, with the accumulation of high-throughput next-generation sequencing (NGS) data, a key goal of systems biology is to construct networks for different cellular levels to explore whole cellular mechanisms. At present, there is no satisfactory method to construct an integrated genetic and epigenetic cellular network (IGECN), which combines NGS omics data with gene regulatory networks (GRNs), microRNAs (miRNAs) regulatory networks, protein-protein interaction networks (PPINs), and epigenetic regulatory networks of methylation using high-throughput NGS data.

**Results:**

We investigated different kinds of NGS omics data to develop a systems biology method to construct an integrated cellular network based on three coupling models that describe genetic regulatory networks, protein–protein interaction networks, microRNA (miRNA) regulatory networks, and methylation regulation. The proposed method was applied to construct IGECNs of gastric cancer and the human immune response to human immunodeficiency virus (HIV) infection, to elucidate human defense response mechanisms. We successfully constructed an IGECN and validated it by using evidence from literature search. The integration of NGS omics data related to transcription regulation, protein-protein interactions, and miRNA and methylation regulation has more predictive power than independent datasets. We found that dysregulation of MIR7 contributes to the initiation and progression of inflammation-induced gastric cancer; dysregulation of MIR9 contributes to HIV-1 infection to hijack CD4+ T cells through dysfunction of the immune and hormone pathways; dysregulation of MIR139-5p, MIRLET7i, and MIR10a contributes to the HIV-1 integration/replication stage; dysregulation of MIR101, MIR141, and MIR152 contributes to the HIV-1 virus assembly and budding mechanisms; dysregulation of MIR302a contributes to not only microvesicle-mediated transfer of miRNAs but also dysfunction of NF-κB signaling pathway in hepatocarcinogenesis.

**Conclusion:**

The coupling dynamic systems of the whole IGECN can allow us to investigate genetic and epigenetic cellular mechanisms via omics data and big database mining, and are useful for further experiments in the field of systems and synthetic biology.

## Background

With advances in molecular biology technologies for whole genome sequencing, expression profiling, and high-throughput experiments, large amounts of biological data covering various biological levels have emerged [1–3]. These kinds of ‘omics’ data include genetic sequences (genomics), microarray-based genome-wide expression profiles (transcriptomics), protein abundance data (proteomics), and microRNA (miRNA) and methylation data, and they provide an unprecedented view of cellular components and their cellular mechanisms in biological systems [4]. With the large amount of genomic/transcriptomic and epigenetic data that has accumulated, a more complex cellular function understanding of living organisms is possible. Computational systems biology techniques able to combine these large, heterogeneous genomic/transcriptomic and epigenetic data sets will provide useful tools to gain additional systems biology insights into cellular mechanism under a specific biological condition, for example, cancer cells or infected cells [5].

Many studies have focused on gene regulatory networks (GRNs) and protein–protein interaction networks (PPINs). Classical graph algorithms have been used to integrate cellular networks of protein–protein and protein–DNA interactions. Well-known simple and complex regulatory circuits have been identified and many putative regulatory circuits have been discussed. In [6], this study includes searches for composite network motifs, which consist of both transcription regulation and protein–protein interactions (PPIs) that recur significantly more often than expected in random networks. In [7], integrated networks comprise transcriptional and PPI data. Interspecific analysis has shown that several types of network motifs are not subject to any particular evolutionary pressure to preserve the corresponding interaction patterns of important biological functions. Studies have also integrated protein–protein and protein–DNA interactions to infer signaling regulatory pathways, but they focus only on pathways that explain gene expression changes in response to gene knockouts [8]. Recently, an integrated cellular network of transcription regulation and PPIs was introduced by *Wang* and *Chen* [9, 10]. They use gene-expression data at multiple time points to prune and combine candidate gene regulatory and signaling networks obtained from genome-scale data. An integrated and focused network for a specific condition of interest is then obtained. The transcriptional network is characterized as a dynamical system in which the expression of a target gene is modeled as a function of the regulatory effect of its corresponding transcription factors (TFs) and mRNA degradation. The modeling of a signaling/protein interaction network accounts for the activity of neighboring loci in the network. Genomic/transcriptomic and high-throughput methods have successfully identified many GRNs and PPINs. However, to explore cellular mechanism, we need more genomic data, such as epigenetic regulation data.

In real cellular systems, the expression of protein-coding genes is controlled by a complex network of genetic and epigenetic regulatory interactions. In addition to the abovementioned genetic regulation mechanisms, epigenetic regulation via DNA methylation is also an important regulatory mechanism in several cellular processes. Recently, high-throughput next-generation sequencing (NGS) data containing information on mRNAs, microRNAs (miRNAs), and methylation has been generated. Therefore, many compelling biological questions center on how regulation and interactions among genes, proteins, and epigenetic regulators give rise to specific cellular mechanisms. To address this problem, we proposed a method to construct an integrated cellular network that can explain specific cellular mechanisms under genetic and epigenetic regulation in response to specific biological conditions, based on the coupling of stochastic dynamic models. Recently, systems biology and computational biology methods have been widely employed to develop stochastic dynamic models that describe biological functions from a dynamic systems perspective [11–23]. Dynamic models to construct an integrated genetic and epigenetic cellular network (IGECN) not only provide a quantitative description of the integrated cellular network, but also predict the cellular mechanism of the network in response to various conditions, gene knockouts, treatments with external agents, etc [24].

**Fig. 1:**
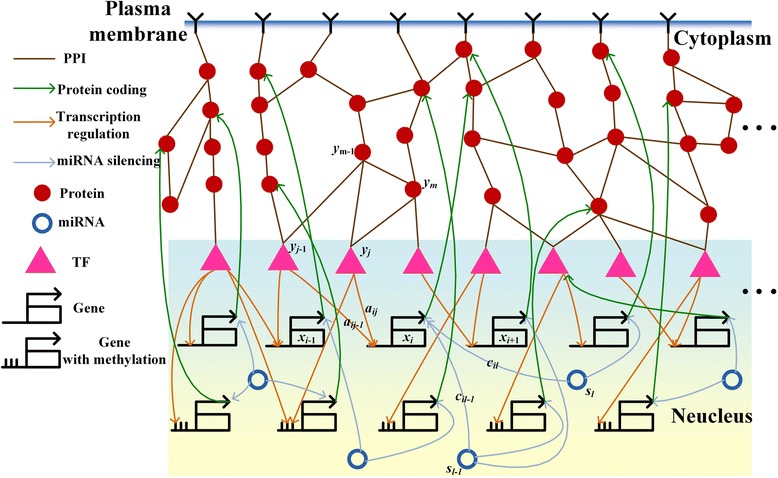
Schematic diagram of candidate integrated genetic and epigenetic cellular networks (IGECN). The candidate IGECN was constructed using omics data and database mining. The candidate IGECN mainly consisted of three sub-networks: the candidate protein-protein interaction network (PPIN) including signal transduction pathways and metabolic pathways, the candidate gene regulatory network (GRN) in which transcriptional regulation occurs, and the candidate miRNA regulatory network. The epigenetic regulation of DNA methylation was considered to influence gene transcription. Transcription factors (TFs) represent the interface between PPINs and GRNs, and genes represent the interface between GRNs, miRNA regulatory networks, PPINs, and methylation regulatory networks

In this study, we integrated omics data, including NGS [25], mRNA and miRNA expression [26], RNA sequencing (RNA-seq) [27], PPIs [28], transcription regulation interaction [29–32], miRNA-target gene association [33–37], and gene ontology (GO) data (http://geneontology.org/) to construct a candidate IGECN. A schematic diagram of the candidate IGECN is shown in Fig. 1. The candidate IGECN mainly consisted of three sub-networks. The first was the candidate PPIN, which included candidate PPIs in signal transduction pathways and metabolic pathways; the second was the GRN, which described transcription regulation; the third was the candidate miRNA regulatory network. Epigenetic DNA methylation was also considered, which involves the modification of DNA to influence mRNA transcription. The TFs in Fig. 1 are at the interface between the PPIN and GRN, and genes are at the interface between the GRN, PPIN, and miRNA and methylation regulatory networks.

Since the candidate IGECN was constructed using omics data and big database mining, for data collected under various experimental conditions (including some that were gathered in vitro), it must contain many false positive interactions and regulations in the candidate PPIN and GRN, respectively. Therefore, it is necessary to prune these false positives, which may not explain real-world cellular mechanisms of biological organisms. In this situation, it is more appealing to evaluate these interactions and regulations in the candidate IGECN for investigating whole cellular mechanisms of a real organism under specific conditions (for example, HIV infections or cancer) using gene expression profile data, protein expression profile data, and miRNA expression profile data via dynamic interaction and regulation models. The false positive interactions and regulations can be pruned based on these real-time data to construct an accurate IGECN for some specific cellular mechanisms.

In this study, three coupling stochastic dynamic models were proposed to develop a candidate IGECN that describes the interplay between the GRN, PPI network, and miRNA network and accounts for methylation regulation in the GRN. After system parameter estimation for coupling stochastic dynamic models of candidate IGECN by these time-profile data, the Akaike information criterion (AIC) and the p-value statistical method [10] were employed to detect the system order (the number of interactions and regulations) of coupling stochastic dynamic models, and then prune the false positive regulations and interactions, which are not significant and out of system order, to obtain the real IGECN for some specific cellular mechanisms in response to some specific biological condition. By the interplay of genetic transcription regulation, PPIs, and miRNA regulation and DNA methylation of the identified IGECN, the cellular machanism can be elucidated comprehensively.

To investigate host-pathogen interspecies cellular interactions and regulations in the host cells is a very important research topic in attempts to understand pathogenic and defensive mechanisms in the HIV infection process and help control the clinic pathogenic infections. In this study, the IGECN is constructed for investigating host-pathogen crosstalks in HIV-infected cells by gene expression profile and miRNA profile of NGS data. Some significant cross-talk hubs in the interspecies IGECN can be extracted as significant crosstalk network marker, from which we could get more insight into the pathogenic mechanism of HIV virus and the defense mechanism of human. Further, the IGECN is also constructed for cancer cells and compared with normal cells to find the significant network marker to investigate some significant cues from the genetic and epigenetic regulatory mechanisms using NGS data.

## Materials and methods for constructing IGECN

### Data selection and processing in gastric cancer and liver cancer

*Kim* and colleagues [26] proposed microarray data, including gene expression data and miRNA expression data, 1 pair of data for normal and tumor tissues of stage I gastric cancer in humans, 10 pairs of data for normal and tumor tissues of stage II gastric cancer in humans, and 15 pairs of data for normal and tumor tissues of stage III gastric cancer in humans. According to The Cancer Genome Atlas (TCGA; https://tcga-data.nci.nih.gov/tcga/), the mRNA and miRNA expression data including 17 dataset for normal tissues and 163 dataset for tumor tissues of stage I liver cancer, 7 dataset for normal tissues and 82 dataset for tumor tissues of stage II liver cancer, 8 dataset for normal tissues and 80 dataset for tumor tissues of stage III liver cancer, and 1 dataset for normal tissues and 6 dataset for tumor tissues of stage IV liver cancer were available. The candidate associations for miRNA-gene regulatory associations are available at the TargetScan [38–41], TF-gene regulatory associations are available at the Human Transcriptional Regulation Interactions database (HTRIdb) [29] and Integrated Transcription Factor Platform (ITFP) [32], and PPIs can be found at The Biological General Repository for Interaction Datasets (BioGRID) [28] and available in the manually curated human signaling network in Edwin Wang Lab website (http://www.cancer-systemsbiology.org/) [42]. In order to integrate the above databases, we use gene symbols in NCBI human gene database to denote the standard names of genes in this study. We used Matlab’s text-file and string manipulation tools in text mining. The interaction candidates were integrated and unified from different databases.

### Data selection and processing in CD4+ T cells of human with HIV-1 infection

*Mohammadi* and colleagues [25] reported gene expression data and miRNA expression data in HIV-1-infected cluster of differentiation 4+ (CD4+) T cells and non-infected (normal) cells to analyze host-pathogen interactions at early (or reverse transcription), middle (integration/replication), and late (virus assembly/budding) infection stages.

### Construction of the dynamic models in IGECN

**Fig. 2:**
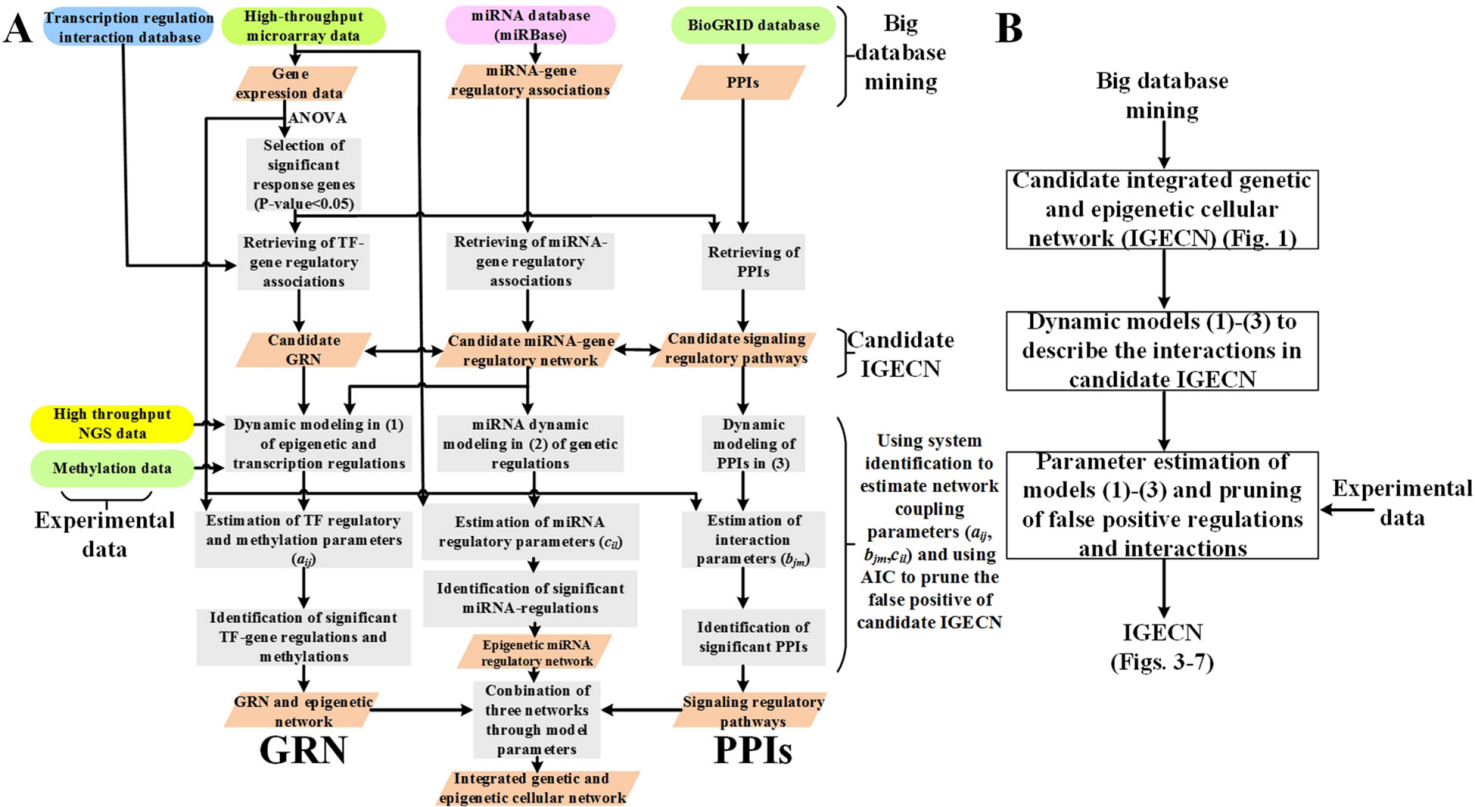
Flowchart (**a**) and block diagram (**b**) of the proposed method to integrate genetic and epigenetic cellular networks

Our goal was to construct an IGECN in which the transcription regulation mechanisms, miRNA regulations, PPIs, and epigenetic regulation via DNA3 methylation are integrated for investigating whole cellular mechanisms under specific biological conditions. A flow chart describing the proposed method to construct the IGECN is shown in Fig. 2. Several kinds of NSG omics data and information derived from databases are integrated, including microarray gene expression data, PPI data, miRNA expression data, and miRNA-target gene association data, as the input for the proposed IGECN construction method. From these data, the candidate GRN, PPIN, and miRNA regulatory network using the broad TF-gene regulation pool, PPI pool, and miRNA regulatory pool, respectively, under various biological conditions were retrieved to construct a candidate IGECN. In this study, NGS data including gene expression and miRNA profiles were used to validate these integrated TF-gene regulation data, PPIs, and miRNA regulation data in the candidate IGECN. Therefore, stochastic dynamic models were used to identify interactions and regulatory mechanisms in the candidate IGCEN by gene expression and miRNA expression profiles using NGS data under a specific biological condition of interest, for example, cancer or infections.

In the transcriptional regulation sub-network of the candidate IGECN, the candidate GRN was depicted as a stochastic dynamic system in which the regulations of TFs and miRNAs are the inputs and gene expression levels of target genes are outputs (Fig. 1). For target gene *i* in the candidate GRN, the stochastic dynamic model is described by the following stochastic discrete dynamic equation:

1 $$ {x}_i\left(t+1\right)={x}_i(t)+{\displaystyle \sum_{j=1}^{J_i}{a}_{ij}{y}_j(t)}-{\displaystyle \sum_{l=1}^{L_i}{c}_{il}{x}_i(t){s}_l(t)}-{\lambda}_i{x}_i(t)+{k}_i+{w}_i(t), $$

where *x*_i_(*t* + 1) denotes the mRNA expression level of the *i*th target gene at time *t* + 1, *a*_*ij*_ represents the regulatory ability of the *j*th TF to the *i*th target gene (with a positive or negative value indicating activation or repression), the regulatory ability *a*_*ij*_ is also influenced by the DNA methylations of the corresponding gene [43], *y*_*j*_(*t*) represents the regulation function of the *j*th TF binding to the target gene *i*, *λ*_*i*_ indicates the degradation effect from the present time *t* to time *t* + 1, *k*_*i*_ represents the basal level (which is also dependent on the methylation of gene *i*), *s*_*l*_(*t*) denotes the expression level of the *l*th miRNA, the parameter *c*_*il*_ represents the rate of miRNA-mRNA coupling (and is dependent on the energy of RNA-RNA binding [44]), and *w*_*i*_(*t*) denotes the stochastic noise due to modeling residue and fluctuation in the expression profile of the target gene. The biological interpretation of equation (1) is that the mRNA expression *x*_*i*_(*t* + 1) of the target gene *i* at time *t* + 1 is a function of the present mRNA expression *x*_*i*_(*t*) and mRNA expression due to transcriptional regulation of *J*_*i*_ TFs that bind to the target gene at time *t*, minus the inhibitory regulation of *L*_*i*_ miRNAs at time *t*, minus the mRNA due to the degradation over time, plus the basal level of mRNA expression, and stochastic noise due to measurement and random fluctuations in the expression profile of target gene *i*.

In the candidate miRNA regulatory sub-network, the stochastic dynamic model of the *l*th miRNA can be described by the following stochastic discrete time dynamic equation [45]:

2 $$ {s}_l\left(t+1\right)={s}_l(t)-{\displaystyle \sum_{i=1}^{I_l}{c}_{il}{s}_l(t){x}_i(t)}-{r}_l{s}_l(t)+{k}_l+{\varpi}_l(t), $$

where the degradation of miRNA, i.e. ${\displaystyle {\sum}_{i=1}^{I_l}{c}_{il}{s}_l(t){x}_i(t)}$, is accompanied by mRNA degradation events induced by the miRNA in (1). That is, the *l*th miRNA induces the degradation of *I*_*l*_ mRNAs at present. *r*_*l*_ denotes the self-degradation rate, *k*_*l*_ denotes the production rate of the *l*th miRNA, and *ϖ*_*l*_(*t*) denotes the stochastic noise.

In the PPI sub-network, the candidate PPIs were depicted as a system in which expressions of mRNA and TFs are input/output of the system (Fig. 1). For a target protein *j* in the candidate PPI sub-network, the stochastic dynamic model of protein expression level is given as follows:

3 $$ {y}_j\left(t+1\right)={y}_j(t)-{\displaystyle \sum_{m=1}^{M_j}{b}_{jm}{y}_j(t){y}_m(t)}+{\alpha}_j{x}_j(t)-{\beta}_j{y}_j(t)+{h}_j+{\zeta}_j(t) $$

where *y*_*j*_(*t*) represents the expression level at time *t* of the target protein *j*, *b*_*jm*_ denotes the interaction ability between the *m*th interactive protein *y*_*m*_(*t*) and the target protein *y*_*j*_(*t*), *α*_*j*_ represents the effect of protein synthesis from mRNA, *x*_*j*_(*t*) denotes the mRNA expression level of the *j*th protein, *h*_*j*_ represents the basal expression level, and *ζ*_*j*_(*t*) is the stochastic noise. The rate of formation of the protein complex *y*_*j*_(*t*)*y*_*m*_(*t*) is proportional to the product of the concentration of each proteins [46, 47], i.e., it is proportional to the probability of molecular collisions between two proteins; thus, the protein interaction complex was modeled as nonlinear multiplication scheme.

The biological interpretation of equation (3) is that the expression level of the target protein *j* at time *t* + 1 is a function of the protein expression level at time *t*, minus protein complex interactions with *M*_*j*_ proteins, plus the translation effect, minus the present protein degradation, plus the basal protein level from other sources and stochastic interactions. It is noted that there is no directionality for interacting proteins in the PPI sub-network.

The regulatory effects and interactions between genes, proteins, miRNA, and DNA methylation in the candidate IGECN are described in the following. Some TFs *y*_*j*_(*t*) at the end of candidate signal transduction pathways (PPINs) regulate target genes according to the regulation function *a*_*ij*_*y*_*j*_(*t*) shown in equation (1), and mRNAs of target genes are also negatively regulated by some miRNAs *s*_*l*_(*t*). The regulated genes influence the corresponding protein expression level via translation from mRNA *x*_*j*_(*t*) to protein *α*_*j*_*x*_*j*_(*t*), as described in (3). An mRNA degradation event, -*c*_*il*_*x*_*i*_(*t*)*s*_*l*_(*t*), described in (1), induced by miRNA *s*_*l*_(*t*), also leads to the degradation of miRNA, -*c*_*il*_*s*_*l*_(*t*)*x*_*i*_(*t*), in (2). Epigenetic DNA methylation also influences the regulation parameter *a*_*ij*_ and basal level *k*_*i*_ in (1) and the translation rate *α*_*j*_ from mRNA to protein in (3). The interplay among genes, proteins, miRNAs, and DNA methylation can be seen in (1)- (3) and Fig. 1, in which TFs are at the interface between PPINs and GRNs, and genes are at the interface between GRNs, miRNA, PPINs, and DNA methylation.

Based on the above stochastic dynamic models (1)- (3), the candidate GRN can be linked through the regulatory parameters *a*_*ij*_ in (1) between genes and their possible regulatory TFs (Fig. 1), and the candidate PPIN can be linked through the interaction parameters *b*_*jm*_ in (3) between possible interacting proteins. The candidate GRN and miRNA regulatory network are linked through the miRNA-mRNA coupling parameters *c*_*il*_ in (1) and (2). Since the candidate IGECN only indicated potential TF-gene regulatory effects, PPIs, and miRNA-mRNA couplings based on data collected by database mining, they should be confirmed by gene expression profile, protein expression profile, and miRNA expression profile in NGS data. The regulatory effects *a*_*ij*_ and *c*_*il*_ in (1), interaction *b*_*jm*_ in (3) and regulation *c*_*il*_ in (2) , which represent the edges of the candidate IGECN, should be identified and validated by empirical gene, protein, and miRNA expression data. These regulatory and interactive parameters were evaluated using temporal data by solving the constrained least square parameter estimation problem. Owing to the limited number of temporal data points, to avoid overfitting when identifying the parameters in (1–3), the cubic spline method [10] was also used to interpolate extra time points for gene expression data. The gene regulatory parameters *a*_*ij*_ and *c*_*il*_ were identified gene by gene (and PPI parameters *b*_*jm*_ protein by protein), so that the candidate integrated network identification process was not limited by the number of genes, proteins, and miRNAs in the candidate IGECN. In other words, the regulatory parameters *a*_*ij*_ and *c*_*il*_ in (1) were first identified for target gene *i* and then for target gene *i* + 1, *i* + 2, etc. The models in (1–3) only represent a small fraction of real biology. For example, many post-transcriptional regulators (RNA-binding proteins) as well as epigenetic regulations beyond methylation were involved. The model uncertainty of real biological system and the fluctuation of expression data were involved in the stochastic noises, *w*_*i*_(*t*) in (1), *ϖ*_*l*_(*t*) in (2), and *ζ*_*j*_(*t*) in (3). A model order detection method such as AIC and statistical assessment like the Student’s *t*-test [10] were used to prune the false-positive regulatory effects and interactions and then detect the true interaction and regulation number of each in the candidate IGECN. AIC [10, 48, 49] was applied to detect the real regulations and interactions by pruning the false positive regulations and interactions in the candidate GRN (1), candidate miRNA regulatory network (2), and the candidate PPI network (3). AIC, which includes both estimated residual error and model complexity in one statistics, quantifies the relative goodness of fit of a model The minimization achieved in AIC will indicate the real network with the true model order. By applying AIC detection method, the insignificant parameters, *a*_*ij*_, *c*_*il*_ and *b*_*jm*_, out of network order are considered as false positives and pruned from candidate IGECNs. We then used Student’s *t*-test to evaluate the significance of each regulatory parameter in (1–3) [10]. In this way, the candidate GRN, candidate PPIN, and candidate miRNA regulatory network based on large-scale data mining were pruned using the temporal data, leading to the construction of an accurate GRN, PPIN, and miRNA regulatory network. Based on the interactive effects between GRNs and signal transduction pathways (or PPINs) via TFs, and among GRNs, miRNA regulatory networks, and PPINs via coupling genes, these three networks were coupled to consist of the IGECN.

We proposed a general method to construct the IGECN using large-scale database mining and system identification for cellular functions. In the following, two examples are given to illustrate the application of the IGECN system to investigate cellular mechanisms under different biological conditions. In the first example, an IGECN was constructed to investigate cellular mechanisms under cancer conditions and in the second example, an IGECN was constructed to investigate pathogenic and defensive cellular mechanisms under HIV infection conditions.

### Identification of the regulatory parameter a_ij_ from the ith TF to the jth target gene, interaction b_jm_ between the jth and mth proteins, and coupling rate c_il_ between the lth miRNA and the ith mRNA in IGECN

After constructing the coupling dynamic models (1)-(3) in the candidate IGECN, the regulative and interactive parameters have to be identified using NGS data. The strategy is to identify the IGECN gene by gene, protein by protein, and miRNA by miRNA. Before applying the identification method, we first examine the dynamic models carefully. In (1), the basal level *k*_*i*_ should be always non-negative, because the gene expressions are always non-negative. Because the parameters in (1) have certain constraints, the regulatory parameters were identified by solving the constrained least square parameter estimation.

The mRNA model (1) was rewritten as the following linear regression form

4 $$ \begin{array}{l}{x}_i\left(t+1\right)=\left[\begin{array}{cccccccc}\hfill {y}_1(t)\hfill & \hfill \cdots \hfill & \hfill {y}_{J_i}(t)\hfill & \hfill {x}_i(t)\hfill & \hfill -{s}_1(t){x}_i(t)\hfill & \hfill \cdots \hfill & \hfill -{s}_{L_i}(t){x}_i(t)\hfill & \hfill 1\hfill \end{array}\right]\left[\begin{array}{c}\hfill {a}_{i1}\hfill \\ {}\hfill \vdots \hfill \\ {}\hfill {a}_{i{J}_i}\hfill \\ {}\hfill \left(1-{\lambda}_i\right)\hfill \\ {}\hfill {c}_{i1}\hfill \\ {}\hfill \vdots \hfill \\ {}\hfill {c}_{i{L}_i}\hfill \\ {}\hfill {k}_i\hfill \end{array}\right]+{w}_i(t)\\ {}\kern3em ={\phi}_i(t){\theta}_i^1+{w}_i(t)\end{array} $$

where *ϕ*_*i*_(*t*) denotes the regression vector which can be obtained from the processing above and *θ*_*i*_^1^ is the parameter vector of target gene *i* to be estimated. In order to avoid overfitting in the parameter estimation process, when identifying the regulatory parameters, the cubic spline method [50–52] was also used to interpolate extra time points for *y*_*i*_(*t*), *x*_*i*_(*t*) and *s*_*i*_(*t*) from NGS data.

The mRNA model (4) at different time points can be arranged as follows.

5 $$ \left[\begin{array}{c}\hfill {x}_i\left({t}_2\right)\hfill \\ {}\hfill {x}_i\left({t}_3\right)\hfill \\ {}\hfill \vdots \hfill \\ {}\hfill {x}_i\left({t}_L\right)\hfill \end{array}\right]=\left[\begin{array}{c}\hfill {\phi}_i\left({t}_1\right)\hfill \\ {}\hfill {\phi}_i\left({t}_2\right)\hfill \\ {}\hfill \vdots \hfill \\ {}\hfill {\phi}_i\left({t}_{L-1}\right)\hfill \end{array}\right]{\theta}_i^1+\left[\begin{array}{c}\hfill {w}_i\left({t}_1\right)\hfill \\ {}\hfill {w}_i\left({t}_3\right)\hfill \\ {}\hfill \vdots \hfill \\ {}\hfill {w}_i\left({t}_{L-1}\right)\hfill \end{array}\right] $$

where *L* is the number of expression time points of NGS data after cubic spline interpolation.

For simplicity, we define the symbols *X*_*i*_, *Φ*_i_, and *W*_*i*_ to express (5) as follows.

6 $$ {X}_i={\Phi}_i{\theta}_i^1+{W}_i $$

The constrained least square parameter estimation problem of *θ*_*i*_^1^ is showed as follows.

7 $$ \underset{\theta_i^1}{ \min }{\left\Vert {\Phi}_i{\theta}_i^1-{X}_i\right\Vert}_2^2\kern0.75em \mathrm{subject}\ \mathrm{t}\mathrm{o}\kern0.5em A{\theta}_i^1\le b $$

where  and *b* = [0^…^0]^T^ that give the constraints to force the miRNA inhibition -*c*_*il*_ to be always non-positive, and the basal level *k*_*i*_ to be always non-negative in (1), i.e. -*c*_*il*_ ≤ 0, and *k*_*i*_ ≥ 0.The constrained least square problem was solved using the active set method for quadratic programming [53, 54].

Similarly, the miRNA model (2) was rewritten in the following regression form.

8 $$ \begin{array}{l}{s}_l\left(t+1\right)=\left[\begin{array}{ccccc}\hfill {s}_l(t)\hfill & \hfill -{s}_l(t){x}_1(t)\hfill & \hfill \cdots \hfill & \hfill -{s}_l(t){x}_{I_l}(t)\hfill & \hfill 1\hfill \end{array}\right]\left[\begin{array}{c}\hfill 1-{r}_l\hfill \\ {}\hfill {c}_{1l}\hfill \\ {}\hfill \vdots \hfill \\ {}\hfill {c}_{I_ll}\hfill \\ {}\hfill {k}_l\hfill \end{array}\right]+{\varpi}_l(t)\\ {}\kern2.75em ={\psi}_l(t){\theta}_l^2+{\varpi}_l(t)\end{array} $$

where *ψ*_*l*_(*t*) denotes the regression vector and *θ*_*l*_^2^ indicates the parameter vector to be estimated. By cubic spline method, at different time points, (8) can be represented as the following equation.

9 $$ {S}_l={\Psi}_l{\theta}_l^2+{W}_l $$

The parameter identification problem is then expressed as follows.

10 $$ \underset{\theta_i^2}{ \min }{\left\Vert {\Psi}_l{\theta}_l^2-{S}_l\right\Vert}_2^2\kern0.5em \mathrm{subject}\ \mathrm{t}\mathrm{o}\kern0.5em B{\theta}_l^1\le b $$

where $$ B=\begin{array}{cc}\hfill \left[\begin{array}{c}\hfill 0\hfill \\ {}\hfill 0\hfill \\ {}\hfill 0\hfill \end{array}\right.\hfill & \hfill \overset{I_l+1}{\overbrace{\left.\begin{array}{ccc}\hfill -1\hfill & \hfill 0\hfill & \hfill 0\hfill \\ {}\hfill 0\hfill & \hfill \ddots \hfill & \hfill 0\hfill \\ {}\hfill 0\hfill & \hfill 0\hfill & \hfill -1\hfill \end{array}\right]}}\hfill \end{array} $$, and *b* = [0^…^0]^T^ that give the constraint to force the miRNA degradation -*c*_*il*_ to be always non-positive, and the basal level *k*_*l*_ to be always non-negative in (2), i.e. -*c*_*il*_ ≤ 0, and *k*_*l*_ ≥ 0. Finally, the protein model (3) uses the same way like above to make sure –*b*_*jm*_ ≤ 0, and *α*_*j*_, *h*_*j*_ ≥ 0. According to large-scale measurement of protein activities, 73 % of the variance in protein abundance could be illustrated by mRNA abundance [55]. mRNA expression levels were frequently used to substitute for the protein expression levels. If the simultaneously measured genome-wide protein expression data and mRNA expression data in each cancer stage are available, the general models in (1–3) can also be applied to identify the real network in cancer. After the parameter identification problem was solved, we can identify the IGECN. For example, we identified the regulatory parameter *a*_RBM5,ETS1_ =-0.22 from the TF ETS1 to the target gene RBM5, interaction parameter *b*_PGR,NCOA2_ =0.06 between the 2 proteins PGR and NCOA2, and coupling rate *c*_USP15,MIR381_ =0.001 between the miRNA MIR381 and the mRNA USP15 in gastric cancer cells.

By using Student’s t-test [56], the p-values for the estimated parameters, including the regulatory ability *a*_*ij*_ in (1), the miRNA-mRNA coupling rate *c*_*il*_ in (1) and (2), the protein interaction ability *b*_*jm*_ in (3), were calculated to determine the significance of the parameters. By using one-way ANOVA, we calculated the p-value for each gene to determine the significance of expression change between normal and tumor cells in cancers, and between normal and HIV-infected human cells. The human DNA methylation profiles of liver cancers (including 41 normal and 369 tumor cells), and gastric cancer (including 2 normal and 396 tumor cells) were available in TCGA. A One-Way ANOVA was also used to calculate the p-value of each gene to determine the significance of DNA methylation change between normal and tumor cells in each cancer.

## Results

The construction of IGECN is very useful to investigate the whole cellular mechanisms of specific biological conditions by big database mining and NGS data. The following two examples were given to illustrate how to construct IGECN to investigate the roles of genetic and epigenetic methylation and miRNA dysregulations in gastric tumorigenesis and HIV infection. Based on these significant core network biomarkers in IGECN, potential multiple drug targets were also proposed for the development of therapeutic treatment.

(I) Construction of IGECN to investigate significant cellular mechanisms in gastric tumorigenesis and hepatocarcinogenesis

Cancer is a complex and heterogeneous disease that is highly robust against therapeutic interventions. It is a constellation of diverse and evolving disorders characterized by the uncontrolled proliferation of cells that may eventually lead to fatal dysfunction of the cellular system. Although some cancer subtypes can be cured by early diagnosis and specific treatment, no effective treatment has been established for many subtypes. Due to the complex, heterogeneous, and evolving nature of cancer, it is appealing to construct an IGECN to gain insight into the carcinogenesis process using large-scale data mining and systematic genetic modeling and various types of omics and temporal data.

**Fig. 3:**
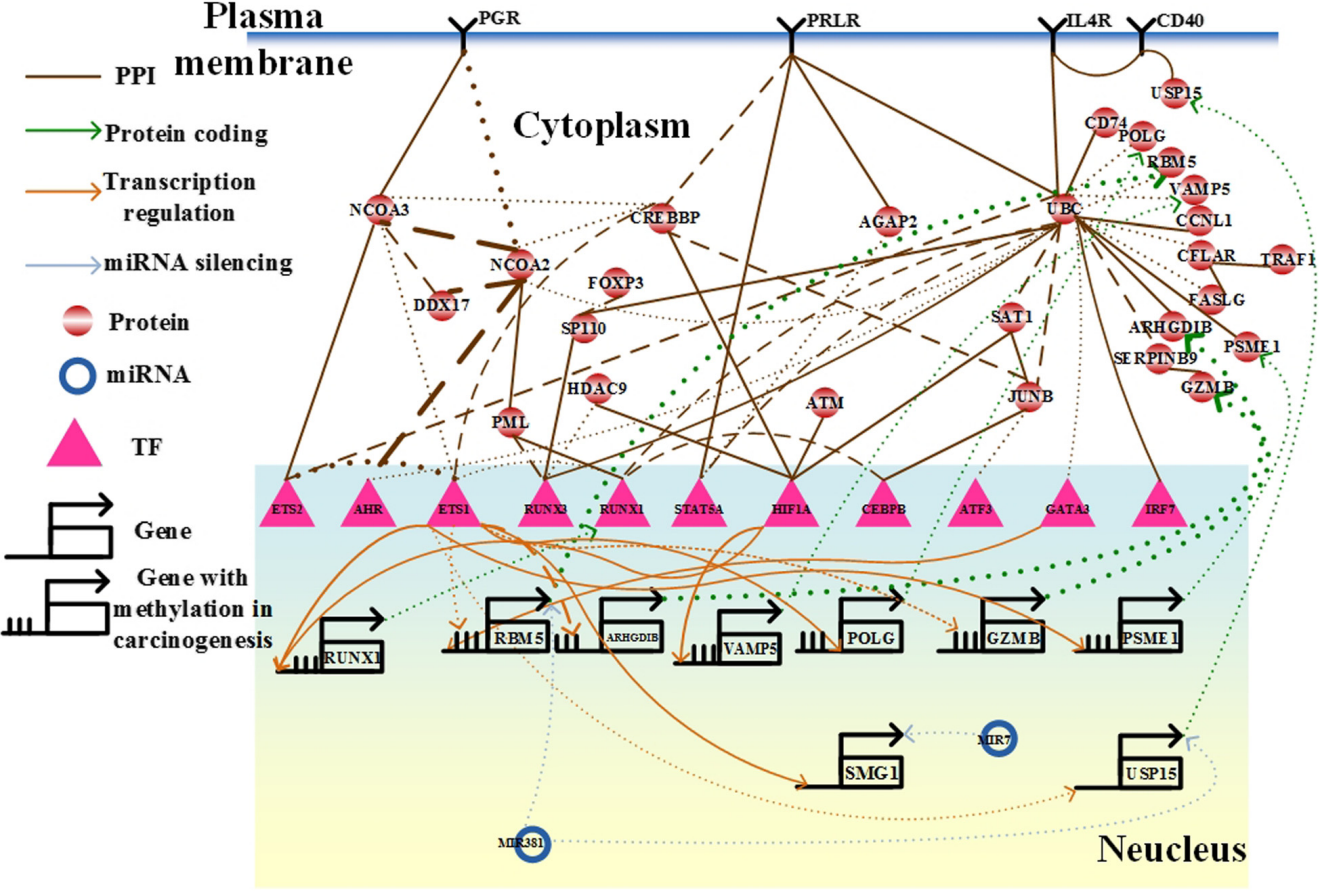
The integrated genetic and epigenetic cellular network for investigating the cellular mechanisms of normal and tumor tissues in gastric cancer. The associations include the networks in tumor cells (*dotted lines*), normal cells (*dashed lines*), and both (*solid lines*). The *bold lines* indicate large genetic and epigenetic regulatory effects or genetic expression. Dysregulation of MIR7 contributes to initiation and progression of inflammation-induced gastric cancer, and PML, a potential drug target, overcomes platinum resistance in platinum-based chemotherapy in gastric cancer

**Fig. 4:**
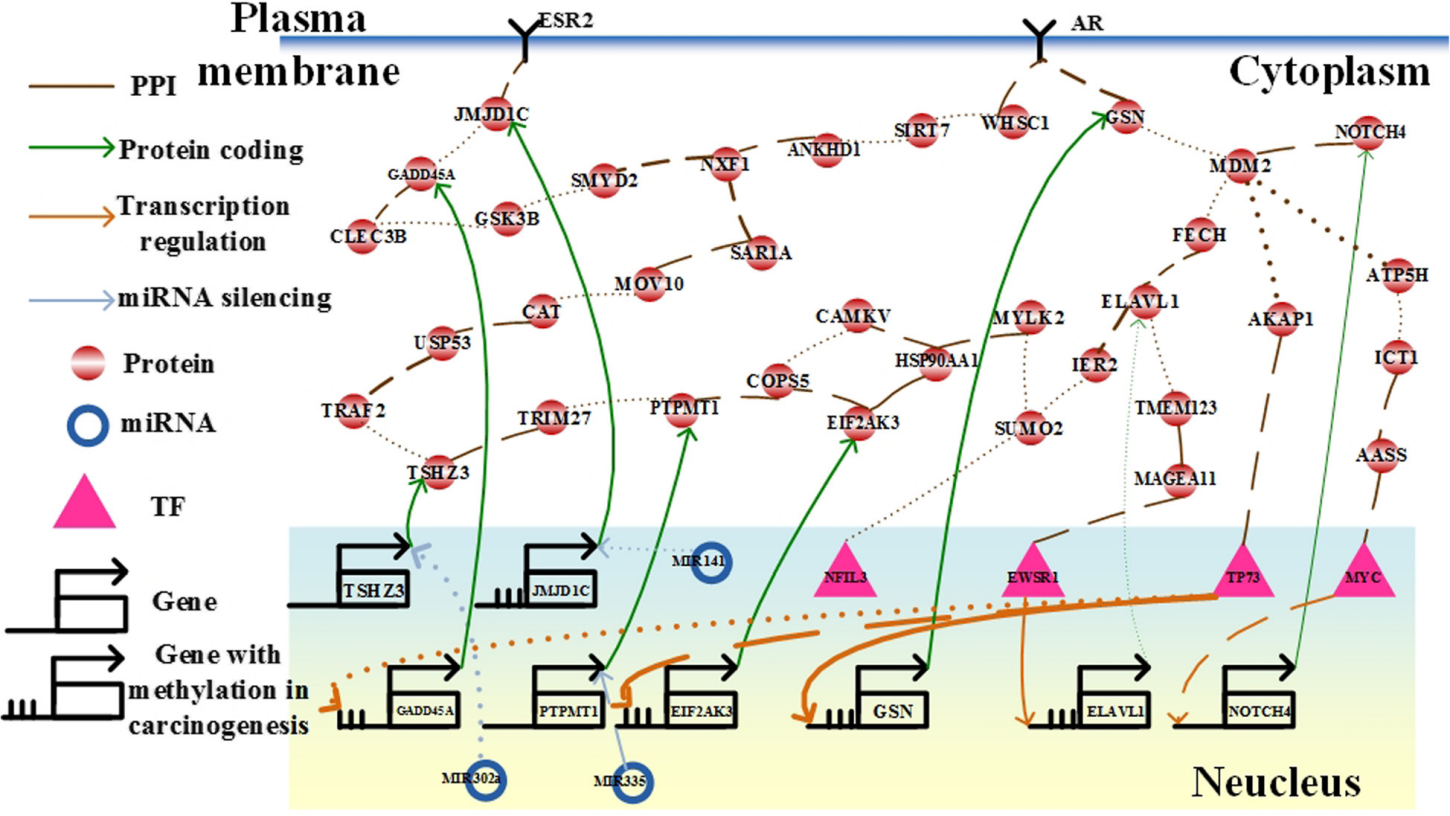
The integrated genetic and epigenetic cellular network for investigating the cellular mechanisms of normal and tumor tissues in liver cancer. The associations include the networks in tumor cells (*dotted lines*), normal cells (*dashed lines*), and both (*solid lines*). The *bold lines* indicate large genetic and epigenetic regulatory effects or genetic expression. Dysregulations of MIR141 and MIR335 contributes to dysfunction of DNA damage response and cell proliferation in human HCC, respectively

Based on the importance of epigenetic modifications in cancer, we identified IGECNs using raw data collected at various stages of cancer as the temporal information for gastric tumorigenesis (Fig. 3) and hepatocarcinogenesis (Fig. 4), demonstrating large differences between miRNA regulations of normal and tumor tissues in gastric tumorigenesis. The results (Fig. 3) show that two cell cycle genes, ubiquitin C (*UBC*) and ataxia telangiectasia mutated (*ATM*), and one chromosomal translocation gene, CREB binding protein (*CREBBP*), play important roles in gastric tumorigenesis via genetic and epigenetic regulation. ATM is an important cell cycle checkpoint kinase [57] , and chromosomal translocation plays a critical role in gastric tumorigenesis [58]. We identified that 7 genes have basal level difference between cancer and normal cells, including *RUNX1* (*P*-value < 1.4☓10^-3^), *ARHGDIB* (*P*-value < 1.6☓10^-2^), *PSME1* (*P*-value < 4☓10^-2^), *VAMP5* (*P*-value < 2☓10^-2^), *POLG* (*P*-value < 1.3☓10^-2^), *GZMB* (*P*-value < 1.44☓10^-2^), and *RBM5* (*P*-value < 8.2☓10^-3^), which are mainly due to DNA methylation of the corresponding genes. In order to validate our finding, we used the epigenomics data of human gastric cancer in TCGA. According to DNA methylation profiles of gastric cancer cells and normal gastric cells from TCGA, we found that 5 genes have methylation change, including *RUNX1* (*P*-value < 3.56☓10^-1^), *ARHGDIB* (*P*-value < 1.81☓10^-1^), *PSME1* (*P*-value < 2.59☓10^-1^), *GZMB* (*P*-value < 1.79☓10^-1^), and *RBM5* (*P*-value < 3.94☓10^-1^), and 2 genes have small methylation change including *VAMP5* (*P*-value < 8.61☓10^-1^), *POLG* (*P*-value < 8.38☓10^-1^) between gastric cancer cells and normal gastric cells (Fig. 3).

Moreover, we also identified that 5 genes have basal level difference between Hepatocellular Carcinomas (HCCs) and normal cells, including *ELAVL1* (*P*-value < 3.69☓10^-5^), *JMJD1C* (*P*-value < 3.59☓10^-11^), *GADD45A* (*P*-value < 7.32☓10^-11^), *EIF2AK3* (*P*-value < 5☓10^-5^), and *GSN* (*P*-value < 1.19☓10^-2^), which is mainly due to DNA methylation of the corresponding genes (Fig. 4). We used the epigenomics data of HCCs in TCGA to support the results. According to DNA methylation profiles of HCCs and normal liver cells from TCGA, we found that 5 gene have methylation changes, including *ELAVL1* (*P*-value < 5.7☓10^-2^), *JMJD1C* (*P*-value < 1.4☓10^-2^), *GADD45A* (*P*-value < 9.6☓10^-2^), *EIF2AK3* (*P*-value < 1.85☓10^-6^), and *GSN* (*P*-value < 8.71☓10^-4^).

(II) Construction of an IGECN for investigating pathogenic and defensive mechanisms of HIV-1-infected cells

Human HIV was first identified in 1983 and rapidly emerged as a virus responsible for a pandemic. At the beginning of this century, the HIV epidemic received global attention, and methods to halt and reverse the AIDS epidemic became an important United National Millennium topic. Currently, approximately 35 million people are living with HIV worldwide. With the advent of highly active antiretrovial therapies, HIV now can be managed as a chronic disease, but developing a cure remains a significant challenge. Therefore, the investigation of interspecific host-pathogen regulatory mechanisms and interactions in cellular genetic and epigenetic systems is a very important research topic to improve understanding of pathogenic and defensive mechanisms in the HIV infection process and to help control clinical pathogenic infections. In this section, using two-sided time-profile HIV-human high-throughput sequence data (NGS), RT-PCR data, miRNA data, and other omic data, the interspecific host-pathogen IGECN was constructed for the HIV infection process. To construct this model, the coupling dynamic equations (1)-(3) should be modified to consider the pathogen in addition to the host species as follows:

11 $$ \begin{array}{c}\hfill {x}_i\left(t+1\right)={x}_i(t)+{\displaystyle \sum_{j=1}^{J_i}{a}_{ij}{y}_j(t)}-{\displaystyle \sum_{l=1}^{L_i}{c}_{il}{x}_i(t){s}_l(t)}+{\displaystyle \sum_{o=1}^{O_i}{d}_{io}{h}_o(t)}-{\lambda}_i{x}_i(t)+{k}_i+{w}_i(t),\hfill \\ {}\hfill {y}_j\left(t+1\right)={y}_j(t)-{\displaystyle \sum_{m=1}^{M_j}{b}_{jm}{y}_j(t){y}_m(t)}-{\displaystyle \sum_{o=1}^{O_j}{g}_{jo}{y}_j(t){h}_o(t)}+{\alpha}_j{x}_j(t)-{\beta}_j{y}_j(t)+{h}_j+{\zeta}_j(t),\hfill \\ {}\hfill {h}_o\left(t+1\right)=h{}_o(t)-{\displaystyle \sum_{j=1}^{J_o}{g}_{jo}{h}_o(t){y}_j(t)}-{\displaystyle \sum_{l=1}^{L_o}{m}_{ol}{h}_o(t){s}_l(t)}-{\kappa}_o{h}_o(t)+{\delta}_o+{\xi}_o(t),\hfill \\ {}\hfill {s}_l\left(t+1\right)={s}_l(t)-{\displaystyle \sum_{i=1}^{I_l}{c}_{il}{s}_l(t){x}_i(t)}-{\displaystyle \sum_{o=1}^{O_l}{m}_{ol}{s}_l(t){h}_o(t)}-{r}_l{s}_l(t)+{k}_l+{\varpi}_l(t),\hfill \end{array} $$

where *h*_*o*_(*t*) denotes expression of the *o*th pathogen protein, and the crosstalk effect of pathogen proteins *h*_*o*_(*t*) are considered. After system identification of coupling IGECN between host and pathogen in (11) by NGS data at different infection stages, we found that 3 genes had differences in their basal levels between cancer and normal cells at the early infection stage, *TAF5* (*P*-value < 2.4☓10^-1^), *DDX3X* (*P*-value < 2☓10^-1^), and *CELF1* (*P*-value < 9.47☓10^-2^); 2 genes had basal level differences between cancer and normal cells at the middle stage, *TAF5* (*P*-value < 5.8☓10^-2^), and *CELF1* (*P*-value < 2.07☓10^-7^); 2 genes had basal level differences between cancer and normal cells at the late stage, *ZNF451* (*P*-value < 9☓10^-2^), and *CELF1* (*P*-value < 1.6☓10^-3^). These genes with different basal levels at different infection stages are mainly due to DNA methylation of stage-specific genes. Previous evidence for DNA methylation, including *TAF5* [59] and *DDX3X* [60] methylation in individuals with HIV infection, *ZNF451* methylation in individuals with Epstein–Barr virus (EBV) infection [61], and *CELF1* methylation in individuals with Moloney leukemia virus infection [62], supports our findings. However, DNA methylation of genes are highly tissue-specific. If the methylation profiles of genes in HIV-infected and mock cells are available in the future, the results can be specifically supported.

**Fig. 5:**
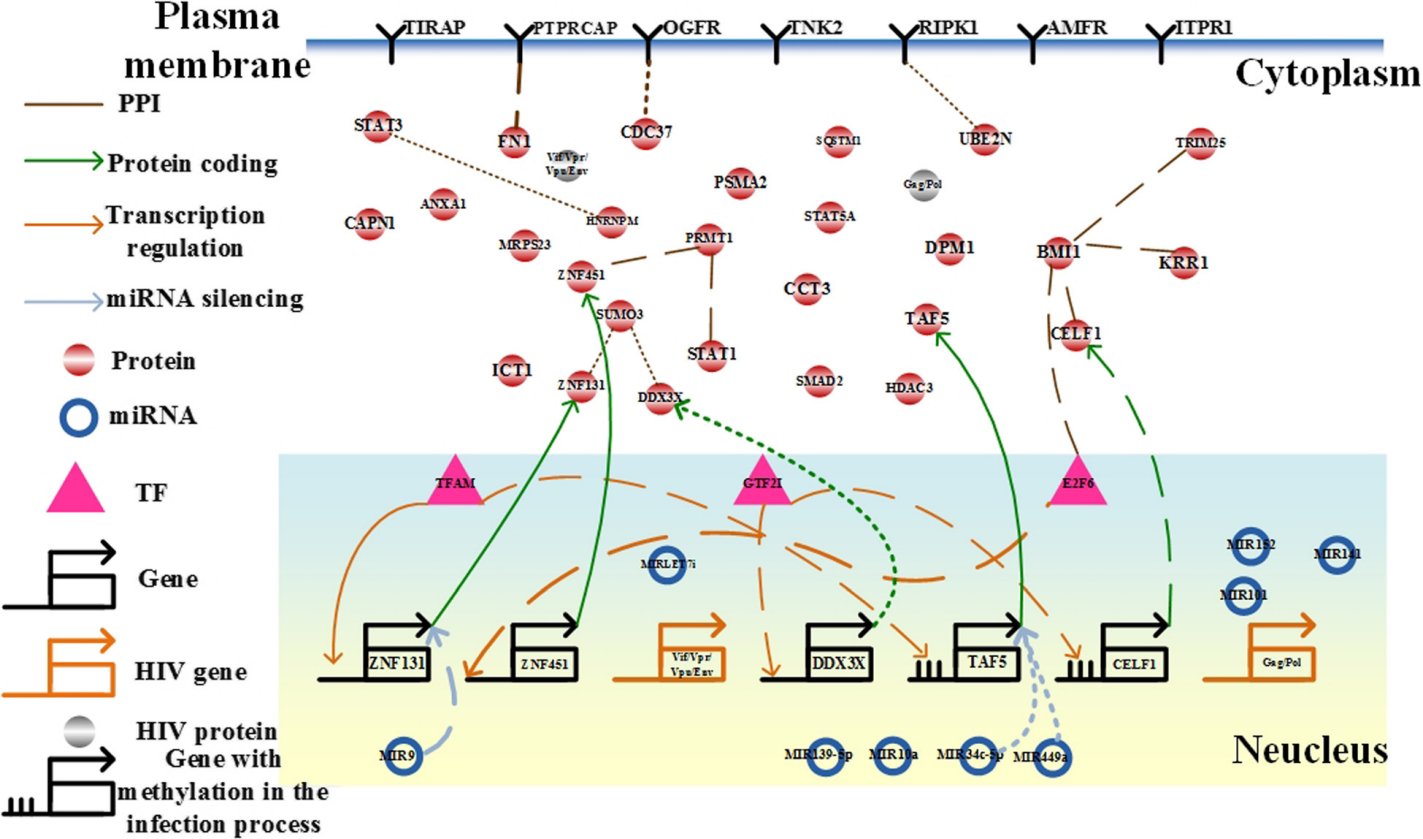
The integrated genetic and epigenetic cellular network for investigating the cellular mechanisms of normal and HIV-infected T cells at the first HIV-1 infection stage, i.e., the reverse transcription stage. The associations include those in HIV-infected cells (*dotted lines*), normal cells (*dashed lines*), and both cell types (*solid lines*). The *bold lines* indicate large genetic and epigenetic regulatory effects or genetic expression. Dysregulation of the signaling cascade from MIR9 to SUMO3 contributes to HIV-1 infection, hijacking CD4+ T cells via dysfunction of the immune and hormone pathways at the early infection stage

**Fig. 6:**
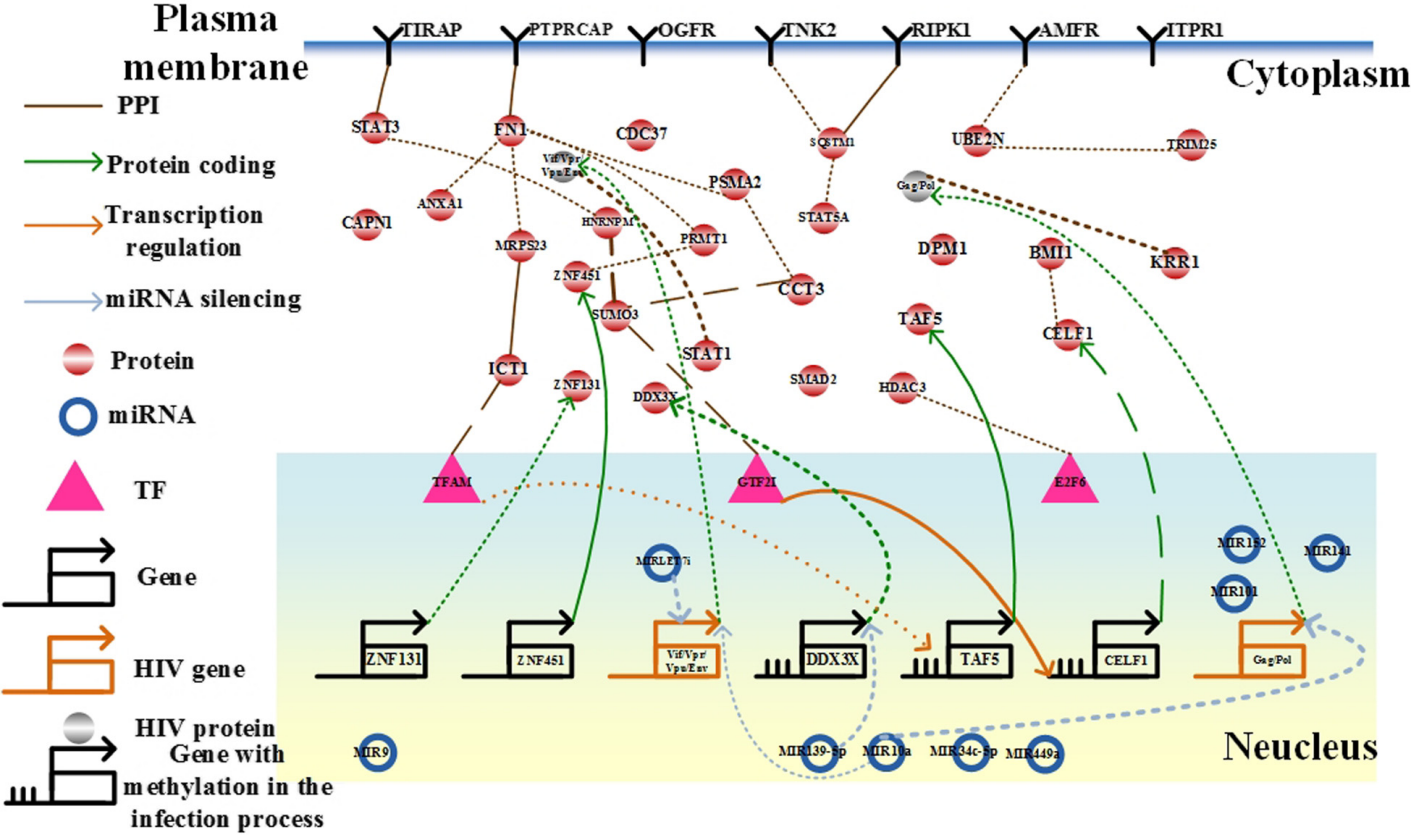
The integrated genetic and epigenetic cellular network for investigating the cellular mechanisms of normal and HIV-infected T cells at the intermediate HIV infection stage, i.e., the integration/replication stage. The associations include those in HIV-infected cells (*dotted lines*), normal cells (*dashed lines*), and both cell types (*solid lines*). The *bold lines* indicate large genetic and epigenetic regulatory effects or genetic expression. Dysregulation of MIR139-5p, and MIRLET7i contributes to dysfunction of the immune response and viral replication via DNA hypermethylation

**Fig. 7:**
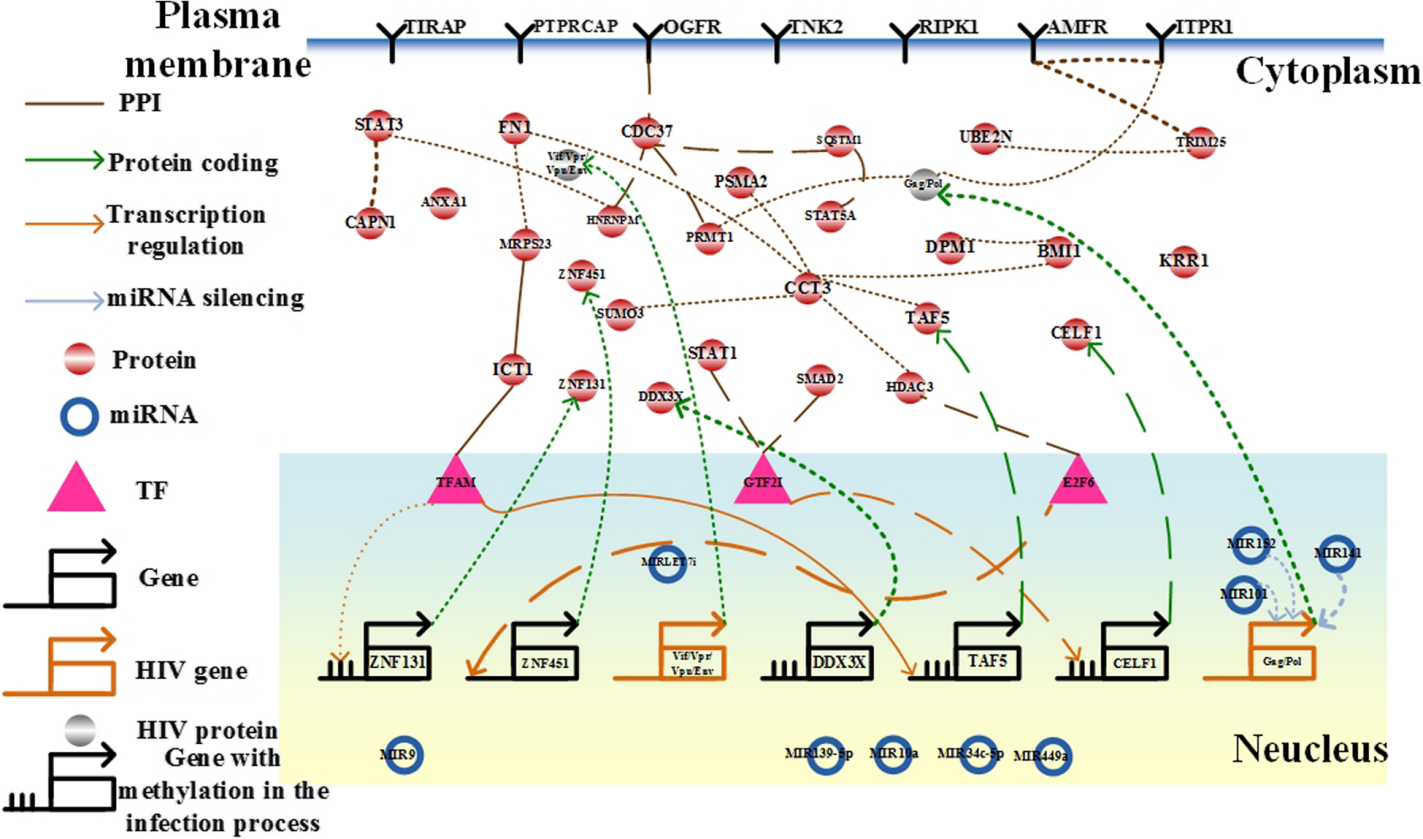
The integrated genetic and epigenetic cellular network for investigating the cellular mechanisms of normal and HIV-infected T cells at the late HIV infection stage, i.e., the virus assembly/budding stage. The associations include those in HIV-infected cells (*dotted lines*), normal cells (*dashed lines*), and both cell types (*solid lines*). The *bold lines* indicate large genetic and epigenetic regulatory effects or genetic expression. Dysregulation of MIR101, MIR141, and MIR152 to the HIV-1 Gag protein contributes to HIV-1 budding and release via DNA hypermethylation, ubiquitin transfer, and endoplasmic reticulum-associated degradation at the late infection stage

By applying the raw expression data and the candidate associations in cancer cells to our model (4), we identified the IGECNs at the three infection stages (Figs. 5, 6 and 7). In the following section, we discuss the contribution of miRNA dysregulation to gastric cancer, HIV-1 infection progress, and liver cancer with respect to biological functions. The results have applications to the development of therapeutic treatments.

Briefly, dysregulation of MIR7 contributes to the initiation and progression of inflammation-induced gastric cancer; dysregulation of MIR9 contributes to HIV-1 infection to hijack CD4+ T cells through dysfunction of the immune and hormone pathways; dysregulation of MIR139-5p, MIRLET7i, and MIR10a contributes to the HIV-1 integration/replication stage through DNA hypermethylation and immune system dysfunction; dysregulation of MIR101, MIR141, and MIR152 contributes to the HIV-1 virus assembly/budding stage through DNA hypermethylation, ubiquitin transfer, and endoplasmic reticulum-associated degradation; dysregulation of MIR302a contributes to not only microvesicle-mediated transfer of miRNAs but also dysfunction of NF-κB signaling pathway in hepatocarcinogenesis.

## Discussion

- Gastric Cancer

### MIR7 contributes to the initiation and progression of inflammation-induced gastric cancer

Until now, it has been suggested that miRNAs play important roles in regulating proliferation and apoptosis to promote the invasion, metastasis, and angiogenesis of gastric cancer cells [63–65]. Although a few connections between genetic-and-epigenetic regulatory mechanisms in gastric cancer cells can be considered independently using biological experiments [63–66], the precise biological functions of miRNAs in gastric cancer cells and miRNAs affecting gastric cancer progression need to be determined by comparing the IGECN with that of normal cells.

In this study, we identified the IGECN including the largest differences in miRNA regulation between normal and cancer cells (Fig. 3). We identified that MIR7 represses SMG1 (*p*-value < 10^-16^) in cancer cells. It has been reported that both MIR7 and SMG1 are involved in the initiation and progression of inflammation-induced gastric cancer [67, 68]. The significant expression changes of MIR7 (*p*-value < 0.151) and SMG1 (*p*-value < 0.009) between normal gastric cells and gastric cancer cells also supported the result that MIR7 and SMG1 play important role in Human gastric carcinogenesis.

We identified that the expression change of MIR381 (*p*-value < 0.83) contributes to the expression changes of RNA-binding protein 5 (RBM5) (*p*-value < 0.008) and ubiquitin specific peptidase 15 (USP15) (*p*-value < 6☓10^-4^) via significant miRNA regulations (*p*-value < 10^-16^) in cancer cells. The activities of RBM5 and USP15 were also significantly activated in gastric cancer. It has been reported that the reduced RBM5 and USP15 expressions result in tumor cell proliferation [69, 70] and induce tumor cell apoptosis [71], respectively. It has also been suggested that dysregulation of two proteins, IL4 and CD40, leads to dysfunction of cell proliferation in mantle cell lymphoma [72], and dysregulation of MIR381 leads to dysfunction of proliferation in esophageal cancer [73]. Thus, we proposed that dysregulation of miRNAs in gastric cancer contributes substantially to the dysfunction of proliferation and apoptosis in gastric cancer via the ubiquitin-proteasome system [74]. TCGA offers a multilayered view of genomics and epigenomics data of many human cancer types. According to DNA methylation profiles of gastric cancer cells and normal gastric cells from TCGA, the methylation changes of GZMB (*p*-value < 0.18), ARHGDIB (*p*-value < 0.18), PSME1 (*p*-value < 0.26), RUNX1 (*p*-value < 0.36), RBM5 (*p*-value < 0.39), POLG (*p*-value < 0.84), and VAMP5 (*p*-value < 0.86) were found by the IGECN. Although the methylation changes of POLG and VAMP5 were insignificant, the gene expression changes of POLG (*p*-value < 0.01) and VAMP5 (*p*-value < 0.02) were still significant. Therefore, we suggested that other regulators may exist in regulating the expressions of POLG and VAMP5 in gastric cancer.

### PML, a potential drug target, overcomes platinum resistance in platinum-based chemotherapy in gastric cancer

Although platinum-based chemotherapy has been used to treat most malignant diseases, platinum resistance still limits its efficacy. The nuclear receptor coactivator family (NCOAs) and the p160 steroid receptor coactivator family (SRCs) in gastric cancer cells exhibit significant differences in interactions observed in the IGECN than in normal cells. It has been proposed that SRC3 plays an important role in intrinsic resistance to platinum therapy in cancers [75]. The results revealed that dysregulation of NCOA2 is transduced by signaling cascades via promyelocytic leukemia (PML) and RUNX1, which is transcriptionally regulated by v-ets erythroblastosis virus E26 oncogene homolog 1 (ETS1). It has been suggested that ETS1 plays an important role in regulating gastric cancer cell invasion, metastasis, and angiogenesis [66, 76]. Because RUNX1 is essential for the development of human hematopoietic stem cells [77], we proposed that PML is a potential drug target that can overcome platinum resistance in the platinum-based chemotherapy in gastric cancer. It has also been suggested that PML mediates glioblastoma resistance to the mammalian target in rapamycin-targeted therapies [78]. Two pharmaceutical drugs, arsenic trioxide and retinoin, potentially target PML to overcome platinum resistance in gastric cancer.

(b) Hepatocellular Carcinoma (HCC)

### Dysregulations of DNA methylation and MIR141 contribute to dysfunction of DNA damage response in HCC

HCC frequently occurs in the context of chronic disease that promotes DNA damage and chromosomal aberrations [79]. Recently, it has been found that the overexpressed MIR141 results in extensive DNA damage [80], and JMJD1C and TP73 are the components of DNA-damage response pathway with implications for tumorigenesis [81, 82]. The accumulated alterations in the DNA-damage response pathway could trigger hepatocarcinogenesis [83, 84]. In this study, we identified that the 3 genetic and epigenetic regulations, including the gain of the miRNA regulation of MIR141 to JMJD1C (*p*-value < 0.789) and the genetic regulation of TP73 to GADD45A (*p*-value < 0.037) in cancer cells, and DNA methylations of JMJD1C and GADD45A, led to not only changes of gene expression profiles of JMJD1C (*p*-value < 7.3☓10^-11^) and GADD45A (*p*-value < 7.3x☓10^-11^) but also changes of DNA methylation profiles of JMJD1C (*p*-value < 0.014) and GADD45A (*p*-value < 0.096) between human normal liver cells and liver cancer cells (Fig. 4). The changes also resulted in the gain of PPI between JMJD1C and GADD45A (*p*-value < 0.48) in hepatocarcinogenesis. Therefore, we suggested that the dysregulations of DNA methylation and MIR141 give rise to dysfunction of DNA damage response in HCC.

### Dysregulation of MIR335 contributes human HCC cell proliferation

Recently, it has been proposed that down-regulation of PTPMT1 is sufficient to trigger cancer cell death [85], and TRIM27 participates in regulating cell proliferation in the development of cancer [86]. We identified that the change in the miRNA expression profile of MIR335 (*p*-value < 0.808) results in the significant change in the gene expression profile of PTPMT1 (*p*-value < 8.4☓10^-4^) between normal liver cells and liver cancer cells (Fig. 4). The expressions of TRIM27 and PTPMT1 were significantly activated in HCC. The significant genetic alterations of TRIM27 (*p*-value < 8.4☓10^-4^) and PTPMT1, and their interaction (*p*-value < 0.32) in HCC promote aberrant proliferation. Therefore, we suggested that dysregulation of MIR335 contributes human HCC cell proliferation.

### D*ysregulation of MIR302a contributes to not only microvesicle-mediated transfer of miRNAs but also dysfunction of NF-κB signaling pathway in hepatocarcinogenesis*

It has been identified that microvesicles transfer miRNA between cells and alter biological functions by affecting signaling pathways in hepatocarcinogenesis [87]. Microvesicles are enriched with maladjusted miRNAs, which could regulate zinc finger proteins. We identified that the change in the miRNA expression profile of MIR302a (*p*-value < 0.734) leads to the change in the gene expression profile of teashirt zinc finger homeobox 3 (TSHZ3) (*p*-value < 0.189), also known as zinc finger protein 537 (ZNF537), between normal liver cells and liver cancer cells (Fig. 4). The expression change of TSHZ3 further contributes to the change in the gene expression profile of TNF receptor-associated factor 2 (TRAF2) (*p*-value < 1.73☓10^-11^), which mediates the dysfunction of the nuclear factor-kappa B (NF-κB) signaling pathway in hepatocarcinogenesis. The dysregulated NF-κB signaling pathway results in HCC angiogenesis and metastasis [88]. Suppression of TRAF2 inhibits the proliferation of several cancer cells [89], and the oncogene in epithelial cancers, TRAF2, contributing to the dysregulated NF-κB signaling pathway has been also identified [89]. Therefore, dysregulation of MIR302a contributes to not only microvesicle-mediated transfer of miRNAs but also dysfunction of NF-κB signaling pathway in hepatocarcinogenesis.

### Dysregulation of the hormone receptors, ESR2 and AR, contributes to hepatocarcinogenesis

The most recent study proposed that menopause, which results from growing estrogen deficiency and physiological aging, increases risk of immune system disorders, mitochondrial dysfunction, cellular senescence, and imbalance between antioxidant formation and oxidative stress [90]. The physiologic and biochemical changes in those functions have a direct effect on liver function and mediate the development of liver disease. In this study, we identified two hormone receptors, estrogen receptor 2 (ESR2) and androgen receptor (AR), which were involved in multiple changes in intracellular signaling cascades. The gene expression profile of ESR2 showed the change (*p*-value < 0.091) between young and old normal liver cells, and the change (*p*-value < 0.063) between young normal liver cells and young liver cancer cells (Fig. 4). The relatively small change in the gene expression profile of ESR2 (*p*-value < 0.45) between old normal liver cells and old liver cancer cells implicates that the gene expression change of ESR2 could be necessary but not sufficient for hepatocarcinogenesis. The significant change in the gene expression profile of AR not only between young normal liver cells and young liver cancer cells (*p*-value < 2.46☓10^-6^) but also between old normal liver cells and old liver cancer cells (*p*-value < 6.13☓10^-7^) implicates that the gene expression change of AR may be required for hepatocarcinogenesis. The result can be supported by the most recent study in hepatocarcinogenesis [91].

(c) HIV-1 Infection

### The signaling cascade from MIR9 to SUMO3 contributes to HIV-1 infection to hijack CD4+ T cells through dysfunction of the immune and hormone pathways at early stage

Owing to the available HIV-1 mRNA gene expression data at middle and late infection stages and the lack of HIV-1 protein expression data, the impact of HIV-1 proteins on the host cells cannot be identified, directly. In this study, we identified the IGECNs at early, middle, and late infection stages (Figs. 5, 6 and 7), including the largest differences in the miRNA regulation and HIV-1 protein interactions at the middle and late stages between normal and infected cells. At early infection stage (Fig. 5), we identified that the expression changes of MIR9 (*p*-value < 0.448) and general transcription factor IIi (GTF2I) (*p*-value < 0.96) contribute to the expression change of small ubiquitin-like modifier 3 (SUMO3) (*p*-value < 0.016) via respectively regulating zinc finger protein 131 (ZNF131) (*p*-value < 9.3☓10^-14^) and DEAD (Asp-Glu-Ala-Asp) box helicase 3, X-linked (DDX3X) (*p*-value < 1☓10^-16^). By comparing miRNA expression profiles between the wildtype HIV-1 infected human CEMx174 lymphocytes and Tat RNA silencing suppressor in HIV-1 infected human CEMx174 lymphocytes [92], Tat RNA silencing suppressor contributed to the expression change of MIR9 (*p*-value < 0.035). It has been also proposed that Tat can induce MIR9 to control inflammatory responses [93], and Tat can also control GTF2I expression during HIV infection [94]. Additionally, it has been also reported that SUMO modification of ZNF131 suppressed estrogen signaling [95], and HIV transcription was suppressed by inducing estrogen signaling [96]. Therefore, we suggested that the HIV infection leads to dysregulation of immune and hormone pathways to hijack CD4+ T cells.

### Dysregulation of MIR34c-5p and MIR449a contributes to the frequency of latent HIV infection

Moreover, we also identified that the expression changes of MIR34c-5p (*p*-value < 0.33), and MIR449a (*p*-value < 0.50) contributed to the expression changes of TATA binding protein-associated factor 5 (TAF5) (*p*-value < 0.25) via miRNA regulations (*p*-value < 1☓10^-16^). It has been suggested that the genetic and epigenetic regulations in TATA protein binding may control the frequency of latent HIV infection [97]. Therefore, we suggested that genetic and epigenetic regulations in TAF5 also contribute to control HIV infection at early stage.

### Dysregulation of MIR139-5p contributes to HIV-1 replication via DNA hypermethylation

At the intermediate HIV infection stage, we identified that the expression change of MIR139-5p (*p*-value < 0.23) contributes to the expression change of DDX3X (*p*-value < 0.14) via miRNA regulation (*p*-value < 1☓10^-16^). The expression of DDX3X was also activated at the intermediate HIV infection stage. Therefore, we suggested that DDX3X could regulate HIV replication through MIR139-5p regulations [98, 99].

### Dysregulation of MIRLET7i contributes to dysfunction of the immune response and viral replication

It has been reported that the HIV-1 Vif/Vpr protein is essential for viral replication [100, 101]. We determined that the Vif/Vpr protein is regulated by MIRLET7i (*p*-value < 1☓10^-16^) and interacts with the human STAT1 protein (*p*-value < 1☓10^-16^) (Fig. 6). Therefore, the expression change of MIRLET7i (*p*-value < 0.079) leads to the expression change of Vif/Vpr protein (*p*-value < 0.318) during the intermediate HIV infection stage. It was supported by the previous observations that MIRLET7i has high homology to HIV-1 sequences (90–100 %) [102] and the immune response of T cells induced by the Vpr-STAT1 interaction was involved in regulating HIV-1 replication [100]. Moreover, by comparing miRNA expression profiles between the wildtype HIV-1 infected human CEMx174 lymphocytes and Vif/Vpr RNA silencing suppressor in HIV-1 infected human CEMx174 lymphocytes [92], Vif/Vpr RNA silencing suppressor contributes to the expression change of MIR7i (*p*-value < 0.107). Therefore, we proposed that the miRNA regulation of MIRLET7i to the Vif/Vpr protein is involved in the induction of the immune response and viral replication (Fig. 6).

### Dysregulation of MIR10a contributes to dysfunction of immune response in CD4+ T cells and viral replication

At the second infection stage, we determined that MIR10a regulates HIV-1 proteins including Gag/Pol (*p*-value < 1☓10^-16^), which significantly interacts with small subunit (SSU) processome component, homolog (yeast) (KRR1) (*p*-value < 1☓10^-16^), also known as HIV-1 Rev binding protein 2 (HRB2) (Fig. 6). Therefore, the expression change of MIR10a (*p*-value < 0.86) contributes to the expression change of Gag/Pol (*p*-value < 0.36). It has been reported that KRR1, an evolutionarily conserved gene, is essential for viral replication [103]. Additionally, MIR10a is involved in the regulation of immune response in CD4+ T cells [104] and coxsackievirus group B type 3 (CVB3) replication [105]. Moreover, it has been shown that the copy number of loci belonging to the MIRLET7 family and MIR10a is correlated with HIV-1 viral load during viral infection [106]. Therefore, we proposed that dysregulation of MIR10a is involved in HIV-1 replication via post-transcriptional regulation of HIV-1.

### Dysregulation of MIR101, MIR141, and MIR152 to HIV-1 budding and releasing through DNA hypermethylation, ubiquitin transfer, and endoplasmic reticulum-associated degradation at the virus assembly/budding infection stage

The final steps of HIV-1 replication are virus assembly and budding. It has been proposed that the HIV-1 Gag protein is involved in the regulation of these steps [107, 108]. At the late infection stage, we determined that regulation of MIR101, MIR141, and MIR152 to Gag (*p*-value < 1☓10^-16^) results in dysfunction of the infected cells through a signaling cascade of 5 proteins, PRMT1, inositol 1,4,5-trisphosphate receptor type 1 (ITPR1), autocrine motility factor receptor (AMFR), tripartite motif containing 25 (TRIM25), and ubiquitin-conjugating enzyme E2N (UBE2N) (Fig. 7). The 4 genes were also highly expressed in HIV-1-infected T cells. Therefore, the expression changes of MIR101, MIR141, and MIR152 (*p*-value < 0.037, *p*-value < 0.108, and *p*-value < 0.126, respectively) contributed to the expression change of Gag (*p*-value < 0.090). We proposed that DNA methylation-associated proteins including PRMT1 and UBE2N contributes to virus release from HIV-1-infected cells to dysregulated uninfected cells through aberrant hypermethylation of host target genes [109]. It has been also suggested that MIR101 [110], and MIR152 [111] induce aberrant DNA hypermethylation in the hepatitis B virus infection. Moreover, ubiquitin transfer is required for efficient HIV-1 release during the final step of viral replication. Therefore, we suggested that the UBE2N-associated ubiquitin transfer is induced by a signaling cascade leading to dysregulation of MIR101, MIR141, and MIR152. Additionally, we also found that ITPR1 is induced to regulate HIV-1 infectivity through endoplasmic reticulum-associated degradation [112].

## Conclusions

In this study, we proposed a new method to construct IGECNs for describing cellular mechanisms by system dynamic modeling and large-scale database mining of NGS data. Furthermore, we applied the method to identify differences in the IGECNs between normal and cancer cells to investigate carcinogenesis, and between HIV-1-infected and non-infected cells to investigate pathogenic and defensive mechanisms at the reverse transcription stage, integration/replication stage, and virus assembly/budding stage based on omics databases and NGS data. Database mining provided all possible candidates for genetic and epigenetic regulatory effects and interactions in the IGECNs related to cellular functions. We used AIC and statistical assessment to prune the false-positive regulatory effects and interactions by applying the dynamic coupling model to NGS data. Finally, we compared the major differences in IGECNs between different biological conditions to identify mechanistic differences to explore the evolution of cellular mechanisms. Based on these network comparisons, we examined how genetic and epigenetic regulation mechanism affects gastric tumorigenesis, hepatocarcinogenesis, and the progression of HIV-1 infections in CD4+ T cells. Therefore, the proposed IGECN construction method allowed us to unravel cellular network mechanisms from genetic and epigenetic mechanisms using omics databases and NGS data. A cancer hallmark network framework has been proposed 9 cancer hallmark networks, including angiogenesis-inducing network, mutating network, dedifferentiation network, EMT (epithelial-mesenchymal transition) network, genome duplication network, immune-escaping network, survival network, metabolic network, and Stroma-network [113]. By comparing the hallmark network difference between normal and cancer cells, cancer clonal evolution and clinical phenotypes, which will have impact on diagnosis and personalized treatment, can be predicted.

## Abbreviations

IGECN: integrated genetic and epigenetic cellular network
NGS: next-generation sequencing
GRNs: gene regulatory networks
PPINs: protein-protein interaction networks
miRNAs: microRNAs
AIDS: acquired immune deficiency syndrome
HIV: human immunodeficiency virus
TFs: transcription factors
RNA-seq: RNA sequencing
GO: gene ontology
AIC: Akaike information criterion
GSEA: gene set enrichment analysis
UBC: ubiquitin C
HCC: hepatocellular carcinoma
USP15: ubiquitin specific peptidase 15
SRCs: p160 steroid receptor coactivator family
NCOAs: nuclear receptor coactivator family
PML: promyelocytic leukemia
ETS1: v-ets erythroblastosis virus E26 oncogene homolog 1
SUMOs: small ubiquitin-related modifier family
PRMT1: protein arginine methyltransferase 1
TCGA: the cancer genome atlas
